# Split‐Mouth Comparison Between Autologous Dentin and Heterologous Bone in Bone Regeneration: Clinical Study on 15 Patients

**DOI:** 10.1002/cre2.70324

**Published:** 2026-03-23

**Authors:** Francesco Ferragina, Angelo R. Sottile, Selene Luccisano, Maria Giulia Cristofaro

**Affiliations:** ^1^ Maxillofacial Surgery Unit, Department of Experimental and Clinical Medicine, Renato Dulbecco Hospital Magna Graecia University of Catanzaro Catanzaro Italy; ^2^ Maxillofacial Surgery Operative Unit, Department of Neurosciences, Reproductive and Odontostomatological Sciences Federico II University of Naples Naples Italy; ^3^ School of Dentistry Magna Graecia University Catanzaro Italy

**Keywords:** autologous bone graft, maxillary atrophy, maxillofacial surgery, split‐mouth clinical trial, Tooth Transformer

## Abstract

**Objectives:**

This study aims to compare the efficacy of heterologous bone grafts to autologous dentine‐derived grafts (processed with the Tooth Transformer) in promoting bone regeneration in patients with posterior maxillary atrophy.

**Materials and Methods:**

A prospective, split‐mouth study was performed on 15 patients (mean age 52 ± 8 years) with symmetrical post‐extraction sites in the jaw. All patients received a heterologous bone graft on one side and an autologous dentine graft on the other. We evaluated new bone formation 4 months after surgery through histomorphometric analysis. Statistical analysis was carried out using paired *t*‐tests and Wilcoxon tests, with a significance level of *p* < 0.05.

**Result:**

A histomorphometric evaluation revealed a significantly higher percentage of new bone formation in the sites treated with autologous dentine (mean 37.68%) compared to heterologous bone (mean 24.05%). The mean discrepancy between the groups was 13.63% (*p* < 0.001). Autologous dentine exhibited superior osteoinductive potential, attributable to the presence of bioactive molecules such as BMPs and Type I collagen, which were absent or denatured in heterologous bone. Both materials provided effective three‐dimensional scaffolding, but dentine showed earlier osteoblastic colonization and enhanced biological activity at the grafting site.

**Conclusion:**

Autologous dentine‐derived grafts processed by the Tooth Transformer significantly outperform heterologous bone in terms of new bone formation in maxillary atrophy. The study highlights the potential of dentine as a promising regenerative biomaterial, offering a safe and effective solution tailored to individual patients, thereby reducing the need for additional donor sites or synthetic alternatives.

## 1 Introduction

Maxillary atrophy, characterized by progressive resorption of the maxillary bone, represents a significant clinical challenge in oral and maxillofacial rehabilitation. This condition often arises from factors such as tooth loss, trauma, congenital abnormalities, or prolonged use of removable dentures (Kämmerer et al. 2023). It affects patients’ functionality, aesthetics, and psychology, thereby negatively impacting quality of life. The loss of both vertical and horizontal bone volume compromises the support for conventional prostheses and complicates the implant placement (Varghese et al. 2023), necessitating advanced surgical procedures and prosthetic solutions.

Recent advances in surgical techniques, biomaterials, and digital planning—such as guided bone regeneration, sinus floor elevation, autogenous bone grafts, alloplastic materials, zygomatic implants, and the use of growth factors like PRF—have significantly expanded treatment options and improved the predictability of maxillary rehabilitation in atrophic cases (Varghese et al. 2023; Choo et al. 2023; Cipollina et al. 2023; Solà Pérez et al. 2022). Each approach has its own advantages and limitations, making it challenging to select the most appropriate technique (Ali et al. 2014). Therefore, treatment planning must be individualized, considering the severity of maxillary atrophy, patient anatomy, overall health, and clinical conditions. The adjunctive use of barrier membranes and growth factors should be considered to optimize bone regeneration when indicated (Qiu et al. 2024; Al‐Aroomi et al. 2024; Mancini et al. 2025).

This study aims to compare the percentage of new bone formation achieved with heterologous bone grafting versus autologous dentine (prepared using the Tooth Transformer device, TT) in a split‐mouth design to minimize inter‐individual variability. A secondary objective is to evaluate the performance of autologous dentin compared to the traditional performance of bone grafts (the historical “gold standard”) through histomorphometric analysis of new bone formation (%NB) at 4 months post‐surgery.

## 2 Materials and Methods

This study was designed as a prospective, randomized, split‐mouth clinical trial. Randomization of the treatment sites (left vs. right side) was performed using a computer‐generated sequence to determine which side received the autologous dentin and which received the heterologous bone. The study included 15 subjects with maxillary atrophy in the posterior regions of the third and fourth quadrants. Each subject had two symmetrical post‐extraction sites: one site was grafted with heterologous bone, the other with autologous dentine. Both sites received a resorbable membrane and tension‐free suturing.

This study was conducted as an exploratory clinical investigation and was not registered in a public trial registry. The study was approved by the University of Catanzaro “Magna Grecia” Ethics Committee (protocol no. 337, dated December 20, 2018) and conducted in accordance with the principles outlined in the Declaration of Helsinki. The full study protocol is available from the corresponding author upon reasonable request.

### 2.1 Inclusion and Exclusion Criteria

Table 1 delineates the inclusion and exclusion criteria for the study.

**Table 1:** Inclusion and exclusion criteria for the study.

Inclusion criteria	Exclusion criteria
– Age > 45 years	– Age < 45 years
– Type D bone atrophy (Misch and Judy 1987).	– Types A, B, B–W, C–W, and C–H bone loss (Misch and Judy 1987).
– Any stage of periodontitis.	– No periodontitis.
– Good oral hygiene.	– Anticoagulant or antiaggregant therapy.
– Willingness to undergo prosthetic surgery.	– Pacemakers.
– Available radiological imaging.	– Diabetes mellitus.
	– Poor oral hygiene (Full Mouth Plaque Index ‐ FMPI > 20%).
	– Patients with uncontrolled periodontitis.
	– Smoking or alcohol abuse.
	– Unwillingness to undergo prosthetic surgery.
	– Incomplete radiological documentation.

### 2.2 Recruitment and Randomization

Patient recruitment was conducted from November 2023 to April 2024. All participants completed the scheduled surgical and follow‐up visits, including histological sampling at 4 months and implant placement at 6 months post‐regeneration.

A priori sample size calculation was not performed due to the exploratory nature of this pilot study. The sample size was determined based on patient availability during the study period and was considered sufficient to provide preliminary data for future adequately powered randomized controlled trials.

Participants were randomly assigned to the test and control sites using a computer‐generated randomization sequence. The randomization process was carried out by an independent investigator who was not involved in the surgical procedures or the assessment of outcomes. The investigator responsible for histomorphometric analysis was unaware of group allocation.

### 2.3 Bone Resorption Classification

We used the Misch and Judy's (1987) classification system to categorize bone resorption:

Type A: substantial bone in all dimensions
Type B: vestibular bone loss
Type B–W: moderate atrophy, reduced crest thickness
Type C–W: advanced atrophy, further thickness loss
Type C–H: significant reduction in both height and thickness
Type D: complete loss of alveolar process, only basal bone remains

### 2.4 Pre‐Surgical Evaluation

Before undergoing the surgical procedure, each patient was required to undergo a pre‐surgical evaluation, which included the following: extra‐oral and intra‐oral analysis, and general systemic examination. Furthermore, a prophylactic antibiotic therapy was administered to each patient.

The general systemic examination is designed to detect concomitant systemic diseases, the use of drugs (e.g., anticoagulant and antiaggregants), the presence of harmful habits (e.g., cigarette smoking, alcohol, and substance abuse), previous cancers of the head and neck region, any adjuvant therapies (e.g., radiotherapy and/or chemotherapy), and the state of pregnancy or lactation.

The extra‐oral analysis enabled the evaluation of the aesthetics of the face, the examination of the soft tissues, and the assessment of the position of the smile line. Conversely, the intraoral analysis enabled the evaluation of various parameters, including dental aesthetics, the overall condition of the dental elements (periodontal, orthodontic, prosthetic, conservative, and endodontic aspects), the level of oral hygiene, and the potential presence of parafunctions.

#### 2.4.1 Surgical Procedures

The surgical procedures were performed under local anesthesia using articaine with adrenaline (40 mg of articaine hydrochloride and 9.10 μg of adrenaline tartrate).

The surgical protocol was meticulously designed to ensure the efficacy of the regenerative intervention, following a standardized sequence of steps.

Initially, dental extraction was performed in the third and fourth quadrant (elements 3.1–3.4 and 4.1–4.4), utilizing a technique aimed at preserving the alveolus. This approach, which has been extensively validated in the extant literature, aims to preserve the post‐extraction bone architecture and to prevent both hard and soft tissue resorption.

Subsequently, the post‐extraction site was subjected to a meticulous decontamination process aimed at eradicating bacterial residues and organic debris, a prerequisite for facilitating optimal tissue regeneration. The biomaterials utilized for the graft were obtained from the same teeth that had been extracted. These have undergone a thorough cleaning process to remove any impurities, followed by processing using a specific apparatus, the TT. Before inserting the extracted tooth into the TT device to obtain an autologous dentin graft/bone deposit, it is essential to perform a thorough mechanical cleaning of the tooth. This step involves the complete removal of tartar residues, soft tissue, caries, pulp, and restorative materials (e.g., fillings, crowns, cements) using high‐speed tools (e.g., diamond cutters) under heavy irrigation. The removal of such contaminants is crucial to reduce bacterial load, limit infectious risk, and prevent the presence of foreign materials in the dentin‐derived biomaterial. Following this thorough cleaning, the tooth can be fragmented and demineralized using a specialized device that produces dentinal granules, which are ideal for promoting bone regeneration (Inchingolo et al. 2023; Minetti et al. 2023). This process facilitates the transformation of dental tissue into a particularly biocompatible autologous material. Following the conclusion of the preparation process, the resulting granular material was inserted into the surgical sites of the third and fourth quadrants. The biomaterials exhibited a high degree of wettability, which facilitates their absorption by the blood. This property has been further enhanced by the addition of autologous growth factors (PRP, PRF) to stimulate bone and soft tissue regeneration. The closure of the surgical site was executed using advanced suturing techniques, employing non‐resorbable polypropylene threads (USP 4|0, EP 1.5), to ensure optimal flap stabilization and promote optimal healing.

In the postoperative period, the site was subject to clinical monitoring with a view to evaluating biomaterials integration and soft tissue healing. Following a period of 7 days, during which the initial healing process occurred, the sutures were removed. Patients were subsequently clinically evaluated at 1, 3, and 6 months. Following this period and once the surgical site had fully healed, the implants were placed. Three months after the regenerative intervention, an orthopantomography was performed, and after 6 months, a Cone Beam Computed Tomography was performed.

During the study period, no major adverse events or complications related to the surgical procedures or biomaterials used were recorded. Post‐operative discomfort and swelling were addressed with standard analgesic therapy, leading to successful resolution without further intervention.

Antibiotic therapy (Amoxicillin 1 g) was administered as a perioperative prophylaxis starting 1 h before surgery, followed by a postoperative regimen for 6 days to prevent infection.

Post‐operative analgesics were prescribed following implant surgery in accordance with current evidence‐based recommendations. As reported in the relevant literature, non‐steroidal anti‐inflammatory drugs, either as a stand‐alone treatment or in combination with paracetamol, are considered the primary approach for managing pain following dental implant procedures. These medications have proven effective in addressing post‐operative pain, particularly during the initial 24–72 h (Khouly et al. 2021). In the present study, analgesic therapy was administered as needed to ensure patient comfort while minimizing unnecessary pharmacological exposure. Patients were instructed on the appropriate use of the prescribed analgesics and monitored for pain resolution during the post‐operative follow‐up.

The bone integration process was evaluated by means of periodic clinical and radiographic examinations. A core biopsy of both grafted areas was performed 4 months after surgery to analyze the histological characteristics of the regenerated bone material. The survey enabled a dual evaluation of the osteointegration process, incorporating both qualitative and quantitative analysis. The primary outcome was the percentage of new vital bone. The patient will be subject to ongoing monitoring, with follow‐up visits scheduled at 3‐month intervals, in accordance with the established protocols for long‐term evaluation.

#### 2.4.2 Tooth Transformer

The TT is a new‐generation medical device designed to convert extracted teeth into autologous biomaterials for bone regeneration. Several clinical and preclinical studies have shown that the dentin particles produced by this system demonstrate remarkable biocompatibility and osteoinduction properties (Inchingolo et al. 2023; Minetti et al. 2023; Ceraulo et al. 2023). This renders TT an innovative alternative to autologous bone grafts or the utilization of heterologous materials. The operation of the device is based on an automated process that combines demineralization and sterilization, reducing the crystallinity of hydroxyapatite, the main mineral component of dentine, and making available morphogenic proteins and growth factors such as bone morphogenic proteins (BMPs) and TGF‐β (Minetti et al. 2023; Minetti et al. 2020, 2024, 2023). These bioactive elements have been demonstrated to stimulate cell adhesion, the proliferation of osteoblasts, and the formation of new bone tissue (Pang et al. 2017; Kim et al. 2010). The patented process is carried out at temperatures below 45°C to preserve the functional integrity of the proteins involved in tissue regeneration. The operational process commences with the cleaning of the extracted teeth, through the removal of restoration materials, endodontic residues, carious cavities, and soft tissues, to guarantee the purity of the final material. Subsequently, the tooth is ground and subjected to a double grinding phase, first at low speed to avoid pulverization and then at high speed. This is followed by manual sieving to obtain particles with controlled granulometry, between 450 and 850 μm. The fragments are subsequently placed within designated sterilizable containers (Bon‐bin), where they are treated with specific solutions for demineralization and sterilization. This process is conducted within a 26‐min cycle that incorporates ultrasonic vibration and exposure to ultraviolet A (UVA) light.

The process is entirely automated, thereby ensuring high levels of standardization, reproducibility, and safety. The utilization of the TT confers substantial clinical benefits, primarily due to the autologous nature of the resulting material, which minimizes the risk of rejection or immune reactions. The automated system facilitates straightforward and predictable preparation procedures. Moreover, the endogenous presence of growth factors renders this biomaterial highly effective in promoting bone regeneration, potentially resulting in reduced healing times when compared to synthetic or heterologous materials. Consequently, TT is regarded as a highly innovative and promising technology in the domain of regenerative surgery, demonstrating the capacity to integrate clinical efficacy, biological safety, and simplicity of use.

#### 2.4.3 Histomorphometry

The histomorphometry analysis was conducted on samples extracted by coring, which were subsequently embedded in methacrylate resin without decalcification to maintain the integrity of the mineral component. The samples were sectioned at varying thicknesses (approximately 30–50 μm) using a hard blade microtome, and then stained with Goldner's trichrome, a specific stain for evaluating mineralized and non‐mineralized bone tissue. The quantitative analysis was carried out using polarized light optical microscopy with an integrated digital acquisition system (e.g., image analysis software ImageJ or equivalent systems). The following areas of interest have been defined: new bone (NB), residual biomaterial (RB), and medullary cavity (MC).

The areas were expressed as a percentage of the total area of the analyzed field (%NB, %RB, and %MC). Measurements were made on at least three sections representative of each sample, in standardized areas, excluding marginal transition zones, to avoid the introduction of artefacts resulting from preparation. The data obtained were utilized to evaluate the quality of bone regeneration and the extent of integration of the biomaterials, with particular emphasis on the continuity between new bone and residual particles, as well as the presence of osteoblasts, osteocyte gaps, and lamellar structure.

Histomorphometric analysis was performed using ImageJ software (version 1.53k; National Institutes of Health, Bethesda, MD, USA) by a blinded examiner.

#### 2.4.4 Statistical Analysis

The descriptive data have been calculated as frequencies (in percentage form, %) and are presented as averages with standard deviations for variables that are normally distributed. The percentages of viable bone in the two groups (heterologous vs. autologous) were compared using a paired *t*‐test. Given that the data are derived from paired measurements (split‐mouth), this test allows for the evaluation of intra‐patient differences. Furthermore, the Wilcoxon test was employed to ascertain statistical significance in a non‐parametric manner. The significance level was set at *p* < 0.05.

The analysis was performed using SPSS 30.0.0 statistical software for Windows (S. Wacker Drive, Chicago, Illinois 60606, USA).

## 3 Results

Fifteen patients, nine of whom were female (60%) and six of whom were male (40%), with a mean age of 52 ± 8 years, completed the follow‐up.

**Figure 1:**
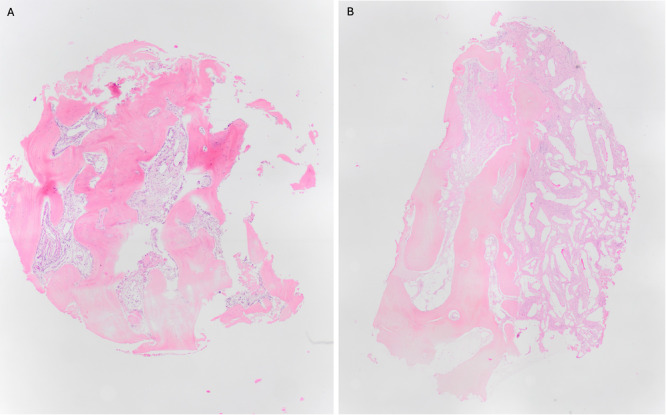
(A) The histomorphometry of a specimen obtained from an autologous dentine graft reveals the presence of well‐formed and compact regenerated bone. (B) The histomorphometry of a specimen obtained from a heterologous bone graft reveals the presence of infiltrated tissue, characterized by red spikes, indicating the presence of biomaterials with inflammatory tissue surrounding them; inside the gaps, there are spheres of biomaterials that are degranulating.

The histomorphometric analysis demonstrated that the heterologous material predominantly functioned as a passive support, without directly inducing the formation of new bone. Conversely, the presence of natural growth factors, such as BMP‐2, TGF‐β, and IGF, trapped in the dentinal matrix of the teeth, induced significant osteogenic activity. New early‐forming bony trabeculates have been observed, even in regions devoid of pre‐existing bone (Figure 1).

An analysis of the parameter of osteoconductivity revealed that both materials exhibited a notable capacity to function as scaffolds, thereby facilitating new bone growth from surrounding bone cells (Table 2). However, the heterologous bone exhibited a predominantly inert behavior, contrasting with the dentine‐derived material, which demonstrated a more dynamic integration, accompanied by evidence of early osteoblastic colonization.

**Table 2:** Histomorphometric parameters considered to assess the osteoconductivity of the two grafts (heterologous vs. autologous).

Features	Heterologous bone graft	Autologous dentin graft
Three‐dimensional scaffolding	Present	Present
Mechanical integration with bone	Good	Good
Volumetric stability	High	High
Colonization by bone cells	Present but slow	Present, early evidence

In consideration of the osteoinduction parameter, which denotes the material's capacity to promote the differentiation of mesenchymal cells into bone cells, autologous grafting exhibited a distinct advantage, as demonstrated in Table 3. Dentin is a natural source of BMPs, type I collagen, and other growth factors. Through a process of enzymatic and physical transformation (Tooth Transformer), these factors are released, thereby stimulating the differentiation of mesenchymal cells into osteoblasts. The heterologous bone, lacking these active components, exhibited no significant osteoinductive properties.

**Table 3:** Histomorphometric parameters considered to assess the osteoinduction of the two grafts (heterologous vs. autologous).

Features	Heterologous bone graft	Autologous dentin graft
Presence of BMPS	Absent (removal during deproteinization)	Present in active form
Presence of type I collagen	Partially denatured residues	Present in high quantity
Biological activity at the grafting site	Limited	High
Spontaneous bone formation	No	Yes (in few cases even without direct contact with native bone)

A quantitative analysis of the data was conducted to assess the percentage of NB 4 months after grafting for both techniques (heterologous vs. autologous), as detailed in Table 4.

**Table 4:** The data represent the percentage of NB found 4 months after grafting for both techniques (heterologous vs. autologous).

Subject	NB (%) in heterologous bone graft	NB (%) in autologous dentin graft	Difference between the two groups (%)
1	26.5	36.6	10.1
2	23.3	33.9	10.6
3	27.2	41.9	14.6
4	31.6	34.6	2.9
5	22.8	31.5	8.7
6	22.8	48.8	26.0
7	31.9	38.6	6.7
8	27.8	40.4	12.6
9	21.7	31.5	9.8
10	26.7	36.7	10.0
11	21.7	40.7	19.0
12	21.7	33.1	11.4
13	25.2	42.3	17.0
14	14.4	36.4	22.0
15	15.4	38.2	22.9

Table 5 provides a comprehensive overview of the primary data collected.

**Table 5:** Comprehensive overview of the primary data collected.

	Heterologous bone graft	Autologous dentin graft
Mean (%)	24.05%	37.68%
Difference (%)	+13.63%	
*p*‐value (paired *t*‐test)	*p*‐value < 0.001	
*p*‐value (Wilcoxon)	*p*‐value < 0.001	

The autologous dentine resulted in an average higher bone formation of 13.63% compared to the heterologous bone. Furthermore, almost all patients exhibited augmented bone regeneration at the site with dentine. This discrepancy is statistically significant, with a *p*‐value less than 0.00000113 (paired *t*‐test) and 0.00006 (Wilcoxon). These *p*‐values indicate an exceedingly low probability that the observed difference is attributable to random fluctuations.

Furthermore, the parametric (paired *t*‐test) and non‐parametric (Wilcoxon) tests yielded congruent results, thereby reinforcing the reliability of the findings.

## 4 Discussion

Bone regeneration is a fundamental strategy to address maxillary atrophy, a condition characterized by the progressive loss of volume and density of the alveolar bone, often resulting from dental loss or periodontal pathologies. This loss of bone can compromise the possibility of dental implants, making surgical techniques aimed at restoring bone tissue necessary (Pérez‐Leal et al. 2023).

Conventionally, bone regeneration has relied on three primary methods: autologous grafts, which are obtained from the patient; heterologous grafts, which are sourced from donors or animals; and synthetic grafts, which are derived from synthetic materials. However, in recent years, the use of dentin as a bone regeneration biomaterial has attracted increasing interest (Um 2018; Kim et al. 2015, 2017). The dentine, the primary component of the tooth located beneath the enamel, exhibits a composition analogous to that of bone, comprising an organic matrix replete with collagen and a mineral component derived from hydroxyapatite.

These characteristics render dentin a biocompatible and osteoinductive material, capable of stimulating the formation of new bone tissue. Furthermore, the utilization of autologous dentine obtained from the patient has been demonstrated to reduce the risk of rejection and disease transmission, thereby offering a safe and effective option for bone regeneration in cases of maxillary atrophy (Um 2018; Mazzucchi et al. 2024; Sun et al. 2024; Cervera‐Maillo et al. 2021).

The findings of the present study suggest that autologous transformed dentine, obtained by employing a Tooth Transformer, results in a significantly higher level of vital bone formation in comparison to heterologous bone. This phenomenon can be attributed to the osteoinductive properties of dentin, which contains extracellular matrix proteins and growth factors (Um 2018; Mazzucchi et al. 2024; Sun et al. 2024; Cervera‐Maillo et al. 2021). Once demineralized, autologous dentin exposes a matrix that is rich in collagen and bioactive proteins, which are similar to those present in bone. This process stimulates the proliferation and differentiation of osteogenic cells, thereby facilitating effective bone regeneration (Khurshid et al. 2024; Barreiro et al. 2023; Li et al. 2013; Lee et al. 2024).

The experimental design, which involved the utilization of a split‐mouth approach, was meticulously devised to mitigate the impact of inter‐individual variability. This methodology entailed the utilization of each patient as their control, thereby ensuring the stringent control of extraneous variables. This approach enhances statistical power and reduces the number of subjects required to achieve substantial results.

Several studies have been conducted to investigate the regenerative capacity of transformed autologous dentin in comparison with conventional grafting materials, such as heterologous bone (López Sacristán et al. 2024; Kuperschlag et al. 2020; Nguyen et al. 2025). Our findings align with those reported in other clinical and preclinical studies.

In a clinical study of 20 patients, López Sacristán et al. compared bone regeneration at sites treated with autologous dentin versus bovine deproteinized bone. The analysis revealed a higher percentage of vital bone in the dentine treated group (mean 41.2% vs. 27.5%, *p* < 0.01), with good integration already at 4 months (López Sacristán et al. 2024). This finding aligns with the results of our study, which documented an average increase of 13.6% in favor of dentine. Instead, a randomized clinical split‐mouth trial conducted by Nguyen et al. in 2025 analyzed the effect of autologous demineralized dentinal matrix in 30 patients after third molar extraction. The sites treated with dentin demonstrated superior bone formation and a substantial reduction in postoperative pain and complications compared to the control group (Nguyen et al. 2025). Several studies have demonstrated that autologous treated dentin exhibits a microarchitectural structure and chemical composition that are strikingly analogous to those of human bone, characterized by robust adhesion and proliferation of osteoblastic cells (Minetti et al. 2023; Inchingolo et al. 2025; Zhang et al. 2021).

This study presents several limitations that should be considered when interpreting the results. First, the relatively small sample size and the exploratory nature of the trial may limit the statistical power and increase the risk of imprecision in the estimated treatment effects. Second, although a split‐mouth design was adopted to reduce inter‐individual variability, the possibility of site‐related or crossover effects between test and control sites cannot be completely excluded. Third, allocation concealment and operator blinding were not feasible due to the nature of the surgical procedures, which may introduce performance bias. However, outcome assessment was performed by a blinded examiner to minimize detection bias. Finally, no adjustment for multiple comparisons was applied, and the findings should therefore be interpreted as preliminary and hypothesis‐generating rather than confirmatory.

## 5 Conclusion

In summary, the findings from the present study corroborate the extant literature regarding the efficacy of autologous dentine as a graft material for bone regeneration. The higher percentage of viable bone observed in dentine‐treated sites, compared to those with heterologous bone, is consistent with data from recent clinical and preclinical studies. Autologous dentine has been demonstrated to be a valid, safe, and effective alternative, capable not only of promoting significant bone regeneration but also of offering relevant clinical benefits such as biocompatibility, autogenicity, and the absence of immune response. Moreover, the utilization of a patient's own tissue offers the potential for cost reduction and streamlined clinical management. The findings indicate that the utilization of dentin might emerge as a promising therapeutic alternative when compared to conventional biomaterials. However, further research involving larger sample sizes and extended follow‐up periods will be necessary to firmly establish this evidence.

## Author Contributions

Conceptualization: Maria Giulia Cristofaro and Francesco Ferragina. Methodology: Francesco Ferragina and Angelo R. Sottile. Software: Angelo R. Sottile. Validation: Maria Giulia Cristofaro and Francesco Ferragina. Formal analysis: Francesco Ferragina. Investigation: Francesco Ferragina and Selene Luccisano. Resources: Selene Luccisano. Data curation: Francesco Ferragina. Writing—original draft preparation: Francesco Ferragina. Writing—review and editing: Maria Giulia Cristofaro and Francesco Ferragina. Visualization: Maria Giulia Cristofaro. Supervision: Maria Giulia Cristofaro and Francesco Ferragina. Project administration: Francesco Ferragina. Funding acquisition: Maria Giulia Cristofaro. All authors have read and agreed to the published version of the manuscript.

## Funding

The authors received no specific funding for this work.

## Ethics Statement

This study was conducted following the ethical standards outlined in the Helsinki Declaration on Medical Protocol and Ethics. The study was approved by the Ethics Committee of Magna Graecia University of Catanzaro (protocol no. 337, dated December 20, 2018).

## Consent

Informed consent was obtained from all subjects involved in the study.

## Conflicts of Interest

The authors declare no conflicts of interest.

## Data Availability

The data are available upon request from the corresponding author, for privacy reasons.
